# Image-Guided Rendering with an Evolutionary Algorithm Based on Cloud Model

**DOI:** 10.1155/2018/4518265

**Published:** 2018-02-19

**Authors:** Tao Wu

**Affiliations:** ^1^School of Information Engineering, Lingnan Normal University, Zhanjiang 524048, China; ^2^Guangdong Engineering and Technological Development Center for E-Learning, Zhanjiang 524048, China

## Abstract

The process of creating nonphotorealistic rendering images and animations can be enjoyable if a useful method is involved. We use an evolutionary algorithm to generate painterly styles of images. Given an input image as the reference target, a cloud model-based evolutionary algorithm that will rerender the target image with nonphotorealistic effects is evolved. The resulting animations have an interesting characteristic in which the target slowly emerges from a set of strokes. A number of experiments are performed, as well as visual comparisons, quantitative comparisons, and user studies. The average scores in normalized feature similarity of standard pixel-wise peak signal-to-noise ratio, mean structural similarity, feature similarity, and gradient similarity based metric are 0.486, 0.628, 0.579, and 0.640, respectively. The average scores in normalized aesthetic measures of Benford's law, fractal dimension, global contrast factor, and Shannon's entropy are 0.630, 0.397, 0.418, and 0.708, respectively. Compared with those of similar method, the average score of the proposed method, except peak signal-to-noise ratio, is higher by approximately 10%. The results suggest that the proposed method can generate appealing images and animations with different styles by choosing different strokes, and it would inspire graphic designers who may be interested in computer-based evolutionary art.

## 1. Introduction

Evolutionary design is an area in which artificial evolution is applied to problems in engineering, design, and the fine arts. One active research topic is the generation of images using evolutionary algorithms [1–7]. A specialized application of evolutionary design is the evolution of nonphotorealistic rendering (NPR) effects [8]. Also, it would be beneficial to researchers to develop and test the evolutionary algorithms for creating interesting and aesthetical images and videos. Generally, there have been a relatively large number of methods on evolutionary algorithms for NPR, and a lot of approaches have been proposed with relative success, but most of these methods fall into user-guided and interactive generation. Recently, some genetic programming based methods have been surfaced and received some attentions [9–12]. Various strokes are introduced into the evolutionary system, such as the line, the starburst, and the triangular stroke, and several aesthetic measures are used to calculate fitness values, including luminance direct match [9–11], deviation from normality, color palette matching, and multiobjective scoring [12, 13]. More recently, a multiagent based art production framework is proposed, which generates images using multiagents with chaotic dynamics features [14]. A methodology is applied for evolving image variants to maximize diversity in image feature metrics [15], and a cooperative coevolution strategy based on the Parisian evolution approach is introduced to produce artistic visual effects from an input image [16]. Still, evolutionary design or evolutionary art presents the opportunity to discover interesting variations of well-known visual styles, as well as completely new ones. The problem of evolving images without human-in-the-loop is still an open issue in evolutionary art; sustained effort is required for autonomous evolutionary art.

Furthermore, uncertainty is an inherent part of evolutionary art. It is easy to describe this process in natural language but difficult to simulate it using computer, and the automatic implementation with uncertainty is still a challenge. We believe that, cloud model, proposed by Li et al. [17, 18], can handle such uncertainty in a better way, since it provides us with more design degrees of freedom and realizes the transformation between a qualitative concept described in words and its numerical representation [19, 20]. Cloud model is a cognitive model between a qualitative concept and its quantitative instantiations and successfully used in various applications. In recent years, cloud model has also been applied in the field of metaheuristics, such as cloud model-based genetic algorithm, cloud model-based evolutionary algorithm, and cloud artificial immune network algorithm [21–24]. By using cloud model, the cloud evolutionary algorithms will have stronger adaptability for the optimization problem and show the perspective of widespread application.

In this context, we propose an image-guided rendering with an evolutionary algorithm based on cloud model (iREC for short), and our intentions are two-fold: (1) How can the image-guided rendering be produced with a cloud model-based evolutionary algorithm? (2) How different types of strokes can affect the aesthetic qualities of the rendered images?

The cloud model-based NPR rendering consists of some steps, including preprocessing an input image by feature extraction, representing the evolution knowledge using cloud model, evolving the individual iteration by iteration and evaluating the fitness function pixel-by-pixel, rendering the canvas on the 2D space provided by the target image, and then generating the result image and animation.

Our method satisfies the CAD property, but what it meant is not the computer aided design, and it stands for cloud model-based evolutionary, automatic evaluation, and dynamic generation: (1) The proposed method involves a novel algorithm using* cloud model-based evolutionary*, although there have been some efforts in using evolutionary algorithm and genetic programming. (2) The proposed fitness-based reproduction for the evolution is with an uncertain measure by the forward and the backward cloud model generators, and the overall procedure is an* automatic evaluation*, but not directly by user interaction. (3) From the artist perspective, the focus is on creativity and iterative process, rather than optimization and the best image, and then the proposed method is a* dynamic generation* by iterations.

In this paper, we make the following contributions. First, we proposed a stroke-based NPR production framework that employs cloud model to achieve the uncertain evolutionary. The algorithm provides the stroke variation of type, color, and orientation. Second, we introduced six types of strokes (both empty and filled) and used the proposed framework to produce artworks. The proposed framework can generate interesting images and animations with the NPR style based on a given input image. Finally, we evaluated the proposed framework with visual comparisons, quantitative comparisons, and user studies to gain insight into the importance and unique affordances of the proposed method. It generally provides the users with the process of generating images, and the immersion effect is also enjoyable.

The remainder of this paper is organized as follows.  Section 2 provides a brief introduction of cloud model. In Section 3, we describe our iREC algorithm in detail: first the preprocessing of the image feature extraction, next the rendering algorithm, then cloud model-based representation, and evolutionary engine; the parameter configuration is also provided in this section. In Section 4, we investigate and suggest eight strokes for rendering, as well as two methods (filled and empty strokes), and then show several examples of the resulting images and animations. Finally, we discuss the results and give some ideas for future improvements in Section 5.

## 2. Cloud Model

Cloud model is the innovation and development of membership function in fuzzy theory and uses probability and mathematical statistics to analyze the uncertainty [18]. In theory, there are several forms of cloud model, successfully used in various applications [17, 18, 25–27]. However, the normal cloud model is commonly used in practice, and its theoretical foundation is the universality of normal distribution and bell membership function [17].

Let *U* be a universe set described by precise numbers and *C* be a qualitative concept related to *U*. Given a number *x* ∈ *U*, which randomly realizes the concept *C*, *x* satisfies *x* ~ *N*(Ex, En^′2^), where En′ ~ *N*(En, He^2^), and the certainty degree of *x* on *C* is as below:

(1) $$\begin{array}{c} \mu \left(\;x\;\right)=\exp\left(\;-\frac{{\left(x-Ex\right)}^{2}}{2{En}^{\prime 2}}\;\right). \end{array}$$

In (1), the expression *z* ~ *N*(*a*, *b*^2^) denotes that *z* satisfies the normal distribution with the mean *a* and the variance *b*^2^. Then the distribution of *x* on *U* is defined as a normal cloud and *x* as a cloud drop.

The overall property of a concept *C*(Ex, En, He) can be represented by three numerical characters of normal cloud model, expected value Ex, entropy En, and hyperentropy He. Ex is the mathematical expectation of the cloud drop distributed in universal set. En is the uncertainty measurement of the qualitative concept, and it is determined by both randomness and fuzziness of the concept. He is the uncertain measurement of entropy, which is determined by randomness and fuzziness of entropy En [17]. It is worth noting that hyperentropy He of a cloud model is a deviation measure from a normal distribution; hence, the distribution of cloud drops can be regarded as a generalized normal distribution.

The kernel of normal cloud model is the transform between qualitative concept and quantitative data, and it is realized by normal cloud generators [17, 26]. On one hand, forward normal cloud generator is the mapping from qualitative concept to quantitative values, and it produces the cloud drops to describe a concept when three numerical characters and the number of cloud drops are input. On the other hand, backward normal cloud generator provides the transformation from quantitative numerical values to quality concept, and a normal cloud model with three numerical characters is defined by computing mean, absolute central moment with the first order, and variance of sample data.

In the normal cloud model, each cloud drop contributes to the concept differently. The Gaussian function satisfies* three sigma rule*, and as a consequence, normal cloud model has 3En* rule*. The majority of cloud drops lie within the interval [Ex − 3En, Ex + 3En], and specifically, cloud drops within the interval [Ex − 0.67En, Ex + 0.67En], called the backbone elements, only account for 22.33% of the universe set but constitute 50% of the cloud model. Then, cloud drops within the interval [Ex − En, Ex + En] make up 33.33% of the universe and constitute 68.26% of the cloud. Similarly, other cases can be also calculated. More information about normal cloud model can be obtained from [17].

## 3. The Proposed Algorithm: iREC

### 3.1. An Overview of iREC

Algorithm 1 outlines the evolutionary process used. Given an input image, the proposed method automatically converts it into NPR style by the following steps:

System initialization (steps (1) and (2)) involves setting parameters, and reading in the source and color target images. Filters such as Sobel, moment, and luminosity are applied to the target image, and the filtered results are saved for future use. Some preprocessing is done, in preparation for fitness evaluation later. This is followed in step (3) by the generation of population based on forward cloud generator, to be used as the initial population for the evolutionary process. Steps (4) to (15) are the main evolutionary loop of the system. Each generation has a different population of cloud model-based representation. First, the fitness values of individuals are calculated in step (6). The processing of an individual begins by initializing the canvas. Using a given pixel selection procedure determined by the parameters *X*_*c*_, *Y*_*c*_, *R*, *θ*, the individual is applied repeatedly to the canvas, resulting in a rendering of the target image. When complete, the canvas is analyzed and evaluated, and the resulting score is assigned as the individual's fitness value. This is repeated for all population members. For each group of individuals, the top *N*_*e*_ elite individuals are determined according to the fitness values of *N*_*p*_ individuals. For each elite individual, we draw a stroke with the *k*th elite individual of the *j*th population according to Algorithm 2. Once all the population has been evaluated, the fitness-based reproduction operators are applied to it, including selection, crossover, and mutation. Then, a cloud model-based reproduction is also conducted, and the gene cloud models are updated using backward normal cloud generator. With the incremental generation *t*, the new individuals are produced according to the forward normal cloud generator, resulting in a new population of individuals. This repeats until best individual is good enough or another stopping criterion is satisfied (see Table 1).

### 3.2. Image Feature Extraction (i Phase)

**Figure 1:**
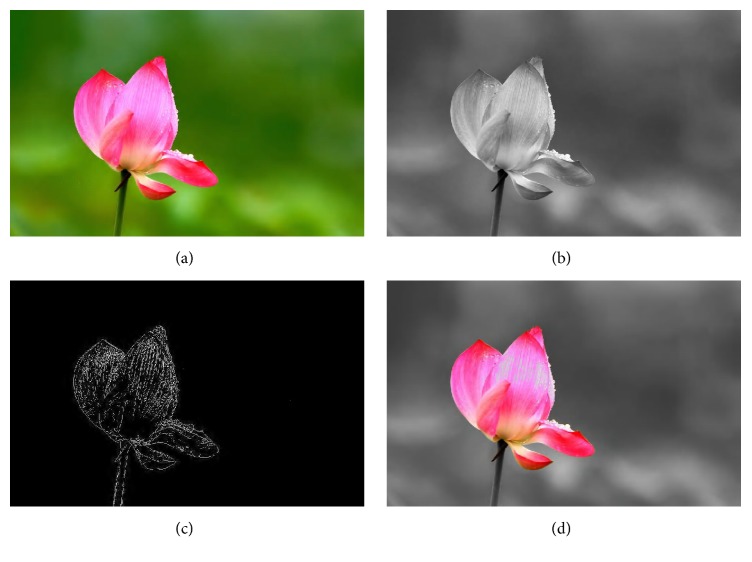
The example image and its feature images: (a) the original image, (b) the grayscale image, (c) the edge image, and (d) the image only with red channel.

Image feature extraction is the preprocessing of the proposed framework, and there are many methods, depending on users' requirement, to solve this problem. In this paper, we use three representative types of feature extraction methods, such as converting color image to grayscale image, edge detection from image, and filtering an image. The result of* i Phase* is denoted as *I*_feature_. The example image and the processed images are shown in Figure 1(a). The grayscale image is obtained by the MATLAB function rgb2gray() applied to the color image. The edge image is obtained by the MATLAB function edge() with a Sobel operator applied to the original image. The filtered image is obtained by extracting red channel of object from the original image. The result images are also listed in Figures 1(b)–1(d), and we use these images as the target in the evolution. Of course, the image feature extraction is still an open problem.

### 3.3. The Rendering Algorithm (R Phase)

#### 3.3.1. Strokes for Painting

To render a stroke on the canvas, we introduce an extensible algorithm, whose input parameters include the center of the circumcircle of the stroke (*X*_*c*_, *Y*_*c*_) and the corresponding circumradius *R*, replaced by *R* and *Rω*_*R*_ for an asymmetrical stroke, where *ω*_*R*_ is a controlling factor on the symmetry of strokes. An opportunity is provided to artist users to select the maximum and the minimum circumradius of the stroke. The algorithm specifies a position (*X*_*c*_, *Y*_*c*_) as the center of the circumcircle of the stroke and then generates an angle *θ* ∈ (0,2*π*) as the skewness of the stroke. The strokes are painted on the canvas with or without the reference of the target image, which will be further discussed in the following section. In addition, it should be noted that the stroke trace is the continuous quantity, while the position of the image pixels is the discrete quantity. In other words, we use the MATLAB function* round*() to approximate this quantity gap between the continuous value of stroke trace and the discrete value of pixel position.

We mathematically define various strokes using a universal expression. Each stroke includes eight components as [*X*_*c*_, *Y*_*c*_, *R*, *θ*, *C*_*r*_, *C*_*g*_, *C*_*b*_, *C*_*a*_]. Specifically, *X*_*c*_, *Y*_*c*_ locate the position of control point of stroke, *R* is related to the size of stroke, and *θ* is the orientation of stroke. *C*_*r*_, *C*_*g*_, *C*_*b*_ define the color of stroke, whose transparency is determined by *C*_*a*_. The remaining parameters *C*_*r*_, *C*_*g*_, *C*_*b*_, *C*_*a*_ are used in the later stage of drawing or filling and further discussed in the following.

*(1) Line Stroke. *We first use four parameters *X*_*c*_, *Y*_*c*_, *R*, *θ* related to the location of strokes. A line can be determined by the center *X*_*c*_, *Y*_*c*_, the length *R*, and the orientation *θ* of the stroke. The discrete point set of line stroke is generated by

(2) X=Xc+Rcos⁡θ,

(3) Y=Yc+Rsin⁡θ.

**Figure 2:**
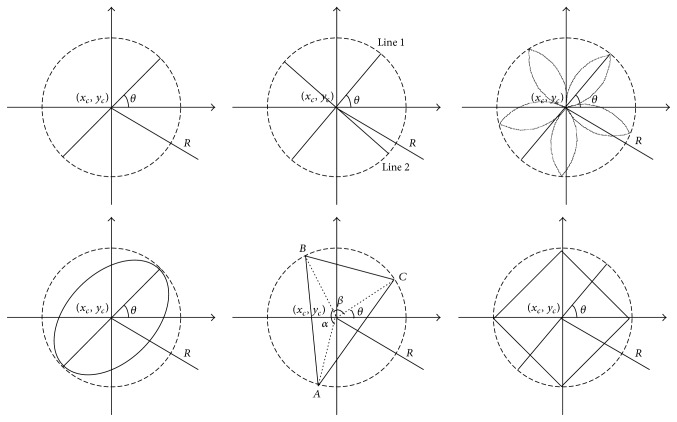
The diagram of strokes, line stroke, crosshair stroke, petal-type stroke, ellipse stroke, triangle stroke, and square stroke, from top to bottom and left to right.

For the reference, Figure 2 gives the diagram of strokes.

*(2) Crosshair Stroke. *It is composed of two perpendicular lines, whose point set is as follows:

(4) X1=Xc+Rcos⁡θ,X2=Xc+Rcos⁡θ+π,

(5) Y1=Yc+Rsin⁡θ,Y2=Yc+Rsin⁡θ+π,

where *α* = 0 : 2*π* denotes the angle of inclination of a line.

*(3) Petal-Type Stroke. *It is like a flower with several petals, and the number of petals is controlled by a random integer *δ* ∈ [3,9]. The discrete point set is

(6) X=Xc+R sin2⁡δθcos⁡θ,

(7) Y=Yc+RωR sin2⁡δθsin⁡θ.

*(4) Circle and Ellipse Stroke. *An ellipse stroke is a generalization of a circle stroke with *ω*_*R*_ = 1. The discrete point set of ellipse stroke is generated by

(8) X=Xc+Rcos⁡αcos⁡θ−RωRsin⁡αsin⁡θ,

(9) Y=Yc+Rcos⁡αsin⁡θ+RωRsin⁡αcos⁡θ,

where *α* = 0 : 2*π* denotes the discretely sampled points from the given ellipse.

*(5) Triangle Stroke. *It is determined by three points *A*, *B*, *C*, whose coordinates are

(10) XC=Xc+Rcos⁡θ,XB=Xc+Rcos⁡θ+β,XA=Xc+Rcos⁡β+α,

(11) YC=Yc+Rsin⁡θ,YB=Yc+Rsin⁡θ+β,YA=Yc+Rsin⁡β+α,

where *β* ∈ (0, *π*/2) and *α* ∈ (0, *π*) denote the random increment related to the vertices of the triangle.

*(6) Square and Rectangle Stroke. *A rectangle stroke is a generalization of a square stroke with *ω*_*R*_ = 1. The discrete point set of rectangle stroke is generated by

(12) X=Xc+dXcos⁡θ−dYsin⁡θ,

(13) Y=Yc+dXsinθ+dYcosθ,

where *d*_*X*_ = [0, *R*, *R*, 0,0], *d*_*Y*_ = [0,0, *Rω*_*R*_, *Rω*_*R*_, 0] denote coordinate values of four vertexes.

For improving the painting, a guided search of color is used with the reference of the target image, and *C*_*r*_, *C*_*g*_, *C*_*b*_, *C*_*a*_ are removed from the draw function of the strokes. Each pixel is painted using the corresponding pixel on the original image. This guarantees that pixels are painted only if they promote convergence towards the target. Conversely, a blind search of color is used without reference to the target image. Thus, *C*_*r*_, *C*_*g*_, *C*_*b*_, *C*_*a*_ should be added into the strokes. In other words, *C*_*r*_, *C*_*g*_, *C*_*b*_, *C*_*a*_ are involved into the evolution process. Obviously, the latter is a modification or variation of the former, and there is almost nothing changed in the algorithm, only additional four parameters are introduced into the evolution. To simplify the problem and facilitate convergence speed, we use the guided search in the paper, and *C*_*r*_, *C*_*g*_, *C*_*b*_, *C*_*a*_ are purposely ignored in the evolution process. We represent each individual using a certain number of strokes. Generally, we mathematically define various strokes using a universal expression, and each stroke includes four components as [*X*_*c*_, *Y*_*c*_, *R*, *θ*], denoted by *s*.

#### 3.3.2. Genetic Representation

The canvas is initialized before each evolution process is executed. There are two options for canvas initialization: (1) copy the target image onto the canvas; (2) initialize the canvas to a given RGB color. With the latter option, the target image's pixel data is still accessible to the evolution process, but not on the canvas itself. In our experiments, we start the evolution process from a white canvas, that is, *I*_res_.

Each stroke can be considered as one chromosome. For each population, the number of individuals is denoted as *N*_*p*_; then we plot each stroke and construct an image with various strokes on the canvas. To confine the strokes in a certain range, we define the bounds of coordinate axis with the height *h* and the width *w*, which is determined by the target image. For the purpose of visual aesthetics, the size of strokes is limited by two constant values *η*_min_, *η*_max_. That is to say, *X*_*c*_ ∈ (0, *w*), *Y*_*c*_ ∈ (0, *h*) and *R* ∈ (*η*_min_*w*, *η*_max_*w*), *Rω*_*R*_ ∈ (*η*_min_*h*, *η*_max_*h*). From the point of view of mathematical formalization, each individual is formed as *I* = [*s*_1_; *s*_2_; …; *s*_*N*_*p*__], where *s*_*i*_ = [*X*_*ci*_, *Y*_*ci*_, *R*_*i*_, *θ*_*i*_] is defined on the above.

Since the position coordinates of pixels are integers, we rasterize the strokes using the round() function, as mentioned above. The used rasterization method is mainly due to two points: First, the too larger stroke will result in an inartistic rendering, the stroke is usually fine, the canvas is relatively larger, and the rounding approximation is rough but acceptable. Second, the accurate calculation is more time-consuming. Of course, the other accurate algorithms are also suitable, even more specially, including Bresenham's line algorithm, the midpoint circle algorithm, and the drawing algorithm of ellipses and Bézier curves, as well as the scanline rendering algorithms for closed regions.

Every selected pixel (*X*_*c*_, *Y*_*c*_) represents the center of a stroke. Therefore, a set of pixels are rendered according to the type of stroke. Each central pixel can result in multiple paint pixels. In addition, it is common for pixels to be rerendered multiple times with the generation, which results in paint mixing effects. The rendering algorithm is described as follows: the current image and the flag buffer are updated from generation to generation. The current image of each generation is as a frame of the final animation. Once the evolution is complete, the current image is as the final result image.

For a selected central pixel (*x*, *y*), the difference between the target image and the temporary image is defined as follows:

(14) $$\begin{array}{c} \mathrm{diff}\left(\;x,y\;\right)=\left|\;I_{feature}\left(\;x,y\;\right)-I_{res}\left(\;x,y\;\right)\;\right|, \end{array}$$

where diff is the difference of intensity for gray image, and then the average difference of three channels if a color image is involved.

To encourage the production of images with clear paint strokes and effectively accelerate convergence, some simple pixel selection strategies were implemented. If the difference diff(*x*, *y*) is lower than a predefined threshold *σ*_diff_ (we fix 3 in the experiment), the painted pixel is acceptable. In addition, a Boolean flag buffer is maintained alongside the canvas buffer. It is first initialized to false. When an individual stroke is applied to a canvas, every pixel colored by it is updated within the pixel set of this stroke according to (2) to (13), respectively. The flag therefore keeps a record of unpainted or unacceptable pixels, which can be used for finding locations of subsequent stroke application. The stroke specifies a position on the canvas, and the updated pixels can be drawn using at least three methods; that is, replace the existing pixels using the stroke pixels, mix pixels by a blending formula, and hold to preserve the existing pixels.

In the following experiments, we use the blending method, and the updated pixels are determined by the canvas values and the corresponding existing pixels, defined as follows:

(15) $$\begin{array}{c} I_{res}=\omega I_{res}+\left(\;1-\omega \;\right)I_{stroke}, \end{array}$$

where *I*_stroke_ denotes a set of pixels related to the current stroke, and we employ *ω* = 0.3.

**Figure 3:**
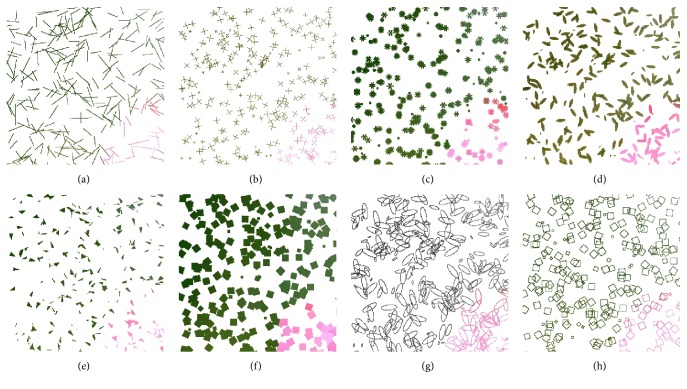
The example strokes: (a) the line stroke, (b) the crosshair stroke, (c) the petal-type stroke, (d) the filled ellipse stroke, (e) the filled triangle stroke, (f) the filled square stroke, (g) the empty ellipse stroke, and (h) the empty square stroke.

The example strokes with 100 iterations are shown in Figure 3, which in order are line, crosshair, petal-type, ellipse, triangle, and square strokes, from left to right, from top to bottom. In addition, empty ellipse and empty square, as other aesthetic styles, are also listed in Figure 3.

### 3.4. Cloud Model-Based Evolutionary Knowledge (C Phase)

**Figure 4:**
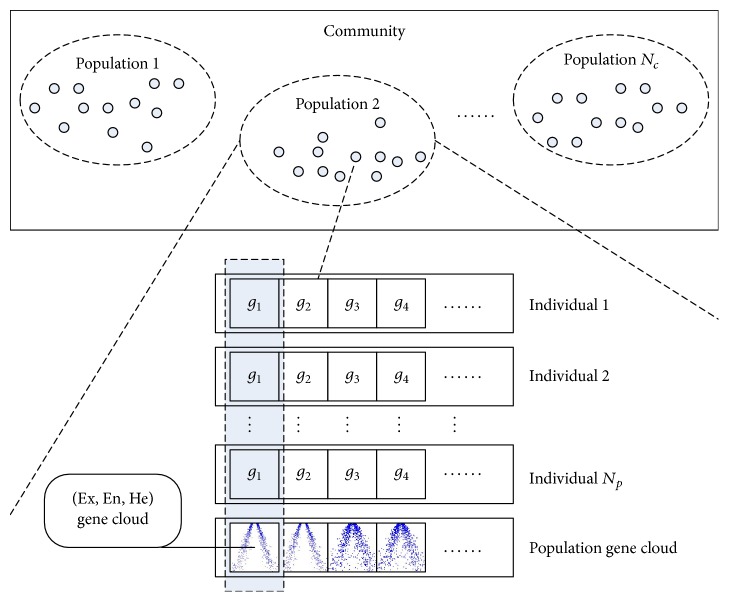
The diagram of evolutionary knowledge.

The overview of evolutionary knowledge based on cloud model is shown in Figure 4. The key idea is gene cloud, which represents each grouping of genes in the community. Each gene cloud corresponds to a component of a set of individuals.

*Gene. *A gene in genetics is a locus or region of DNA that encodes a functional RNA or protein product and is the molecular unit of heredity. In this paper, a gene is as one of the attributions of the stroke, and we use four attributions, *x*_*c*_, *y*_*c*_, *R*, *θ*, as shown in Figure 2. Therefore, the genes are arranged in four groups (see Figure 4), with each group of four genes defining a stroke to be drawn on the image. Furthermore, some additional attributions can be also introduced to improve the aesthetics of result images and animations, for example, the gray level for the grayscale images and the number of the petals for the petal-shaped stroke.

*Individual. *In iREC, each grouping of genes is treated as an individual, in which the integers *X*_*c*_ and *Y*_*c*_ denote the center of the circumcircle of the stroke, while the floating numbers *R* and *θ* define the radius and the skewness of the circumcircle of the stroke.

*Population. *A population is a summation of all the individuals of the same group, which are a part of particular cloud drops, defined by the population gene cloud, as described below. The number of the population individuals in a community is called the population size, denoted by *N*_*p*_.

*Elite Individual. *Population elite denotes the position of the optimal individual, namely, *P*_best_, and the population elite will be saved and in the corresponding position a stroke will be drawn in the following rendering phase. The number of elite individuals in a population is called the elite size, denoted by *N*_*e*_.

*Community. *A community is an iterative unit of a fixed size, which consists of several groups of populations connected by interbreeding relations. The number of the population groups in a community is called the community size, denoted by *N*_*c*_.

*Gene Cloud. *Each grouping of genes in the community is represented by a cloud model, namely, gene cloud *g*_*i*_(Ex_*i*_, En_*i*_, He_*i*_), where *i* represents the *i*th gene and Ex_*i*_, En_*i*_, He_*i*_ are three digital characteristics of cloud model. Each gene is produced by a gene cloud, using a forward normal cloud generator [17], during the evolution.

*Population Gene Cloud. *For a community, a set of cloud models is generated to represent the distribution characteristics of the same dimension of all individuals, called population gene cloud [*g*_1_^*j*^, *g*_2_^*j*^, *g*_3_^*j*^, *g*_4_^*j*^], where *j* represents the *j*th population. That is to say, a community is determined by the population gene clouds, which is a cloud matrix as below:

(16) $$\begin{array}{c} \left[\begin{array}{ccccc} g_{1}^{1}\left({Ex}_{1}^{1},{En}_{1}^{1},{He}_{1}^{1}\right) & \cdots & g_{i}^{1}\left({Ex}_{i}^{1},{En}_{i}^{1},{He}_{i}^{1}\right) & \cdots & g_{4}^{1}\left({Ex}_{4}^{1},{En}_{4}^{1},{He}_{4}^{1}\right)\\ \vdots & \cdots & \vdots & \cdots & \vdots \\ g_{1}^{j}\left({Ex}_{1}^{j},{En}_{1}^{j},{He}_{1}^{j}\right) & \cdots & g_{i}^{j}\left({Ex}_{i}^{j},{En}_{i}^{j},{He}_{i}^{j}\right) & \cdots & g_{4}^{j}\left({Ex}_{4}^{j},{En}_{4}^{j},{He}_{4}^{j}\right)\\ \vdots & \cdots & \vdots & \cdots & \vdots \\ g_{1}^{Nc}\left({Ex}_{1}^{Nc},{En}_{1}^{Nc},{He}_{1}^{Nc}\right) & \cdots & g_{i}^{Nc}\left({Ex}_{i}^{Nc},{En}_{i}^{Nc},{He}_{i}^{Nc}\right) & \cdots & g_{4}^{Nc}\left({Ex}_{4}^{Nc},{En}_{4}^{Nc},{He}_{4}^{Nc}\right) \end{array}\right]. \end{array}$$

Individual is the basic unit of evolutionary in the classical methods; in contrast, there is more than one population in our iREC, and the evolutionary unit is the population. An individual gene *v*_*i*,*k*_^*j*^ denotes the *i*th gene component from the *k*th individual and the *j*th population.

Therefore, considering each individual as a stroke, each component of the cloud model determined by the individuals can be considered as the population gene cloud. That is, the three numerical characters on *X*_*c*_ are calculated by the backward cloud generator from the sequence {*X*_*ci*_∣*i* = 1,2,…, *N*_*p*_}. The other components *Y*_*c*_, *R*, *θ* are processed in the same way.

### 3.5. Cloud Model-Based Evolutionary Engine (E Phase)

*Fitness. *Fitness function denotes adaptation degree of each individual aimed at their located environment in the community. It is used to evaluate the individual and decide which individual to retain or eliminate. In this paper, fitness function is the expression of the difference between the target image and the temporary image and is defined as follows:

(17) diffI=∑x∈1,w ∑y∈1,hdiff⁡x,y,f=11+diffI,

where diff_*I*_ is the sum of the difference pixel-by-pixel according to (14).

*Initialization. *The population size depends on the nature of the problem but typically contains several hundreds or thousands of possible solutions. Often, the initial population is generated randomly, allowing the entire range of possible solutions (the search space). In our method, the initial individuals are generated by the forward normal cloud generator, whose numerical characters are determined randomly.

*Selection. *In this stage, potentially useful solutions are selected for recombination. In iREC, the selection includes population selection and individual selection. Firstly, the fitness function is evaluated for each individual in each population, providing fitness values, which are then normalized. Normalization means dividing the fitness value of each individual by the sum of all fitness values, so that the sum of all resulting fitness values equals 1:

(18) $$\begin{array}{c} \text{fitness}=\frac{f-f_{worst}}{f_{best}-f_{worst}}, \end{array}$$

where *f* is the fitness value of the processed individual and *f*_worst_ and *f*_best_ denote the worst and the best fitness values in the population.

For individual selection, we use roulette wheel selection. The population is sorted by descending fitness values. Accumulated normalized fitness values are computed; the accumulated fitness value of an individual is the sum of its own fitness value plus the fitness values of all the previous individuals. A random number *N*_*r*_ = rand⁡(1) between 0 and 1 is chosen. The selected individual is the first one whose accumulated normalized value is greater than *N*_*r*_.

There are two strategies of population selection. Population unified selection strategy is to select the elite individuals from all the populations and then assign them to each population as the seeds. Meanwhile, population independent selection strategy is the selection of elite individuals from each population, which are as the seeds in the next generation. The convergence rate of the former strategy is faster, while the latter one can well ensure the diversity of individuals. According to the needs of the aesthetic requirements, we used the population independent selection strategy.

*Crossover. *It is a process of taking more than one parent solution and producing a child solution from them. In iREC, crossover operation can only occur between two individuals from different populations. That is, the operator essentially acts on the population, but not individual. Crossover only occurs at a particular mating according to a predefined crossover probability *P*_*c*_. There are many crossover techniques, and we use the arithmetic mean value of the parent gene:

(19) vi,kj=αcvi,kj+1−αcvi,k′j′,vi,k′j′=αcvi,k′j′+1−αcvi,kj,

where *α*_*c*_ ∈ (0,1) is a random number, namely, the crossover coefficient.

*Mutation. *This alters one or more gene values in a chromosome from its initial state. The selection rule remains the same for both crossover and mutation. Mutation only occurs according to a predefined mutation probability *P*_*m*_, and this probability should be set low:

(20) $$\begin{array}{c} v_{i,k}^{j}=\left\{\begin{array}{cc} v_{i,k}^{j}+\left(u_{k}-v_{i,k}^{j}\right)\beta_{m}^{1-t/G_{m}} & \beta_{m}\mathrm{mod}2=1,\\ v_{i,k}^{j}-\left(v_{i,k}^{j}-l_{k}\right)\beta_{m}^{1-t/G_{m}} & otherwise, \end{array}\right. \end{array}$$

where *G*_*m*_ is the maximum generation and *β*_*m*_ is a random integer, namely, the mutation coefficient.

*Update. *In this operator, all the individuals are removed, the numerical characteristics of each gene cloud model are calculated by the backward cloud generator and then the individuals of the new generation are reproduced by population gene cloud. Compared with the traditional evolution operators, our method involves a cloud generator between qualitative concept and quantitative data.

*Pixel Ratio. *We refer to the proportion of the matched image pixels, whose value is true in the flag buffer *I*_flg_. The mathematical expression of the matched pixels can be

(21) $$\begin{array}{c} \frac{\sum_{x\in \left[1,w\right]}\sum_{y\in \left[1,h\right]}I_{flg}\left(x,y\right)}{hw}\times 100\%. \end{array}$$

**Figure 5:**
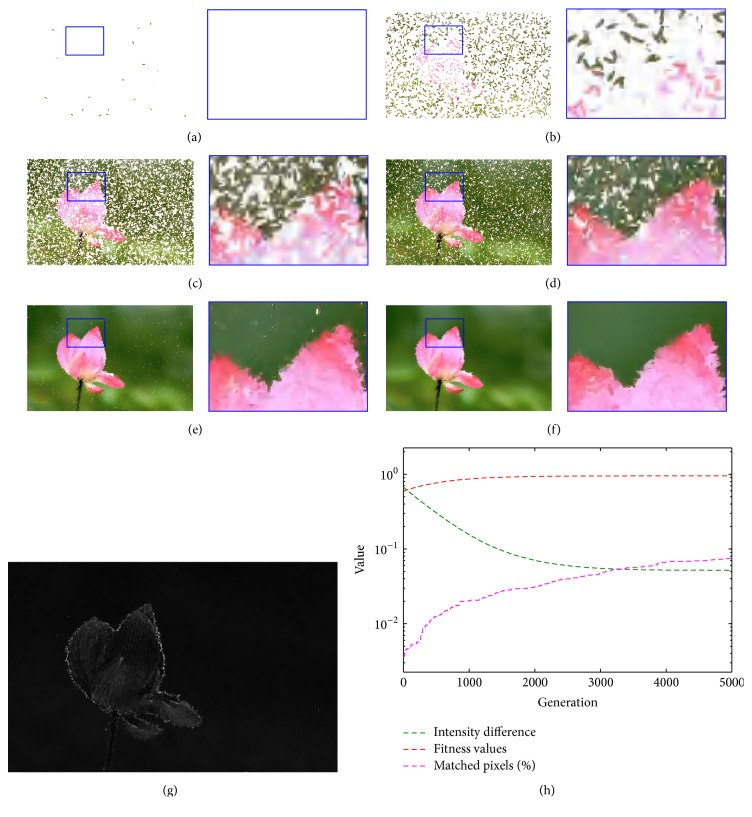
Sequence from a run of the evolution by using filled ellipse stroke. (a)–(f) The buffer images with the generation of 1, 100, 500, 1000, 3000, and 5000; each subfigure is composed of the result and its enlarged portion marked with blue, (g) the intensity difference between the target image and the result image, and (h) the quantitative results on intensity difference, fitness values, and matched pixels in the evolution.

Figures 5(a)–5(f) show a sequence from an animated movie showing the target with a series of frames of the subject gradually emerging, as well as the enlarged portion marked with blue rectangle. The system starts with a white canvas, and more and more strokes are placed onto the canvas; then an aesthetic image is generated iteration by iteration. During the evolution, the flower is more and more clear, and the number of white pixels becomes lower and lower. For the reference, the video result (MP4 format) is attached in the Supplementary Materials available online (available here).

The intensity difference between the target image and the result image is listed in Figure 5(g). There are a lot of black pixels in the difference image. In other words, the final result is rather closer to the target image. Only pixels around the edge appear white and need to be improved. In fact, this is just the charm and interesting part of the proposed method, since the purpose of our method is to provide a NPR result, rather than the original image.  Figure 5(h) shows the progression of fitness values, the proportion of matched pixels, and the intensity difference during the course of the evaluations for one run. Both of the curves of fitness values and matched pixels produce similar tendency with the various generations. The corresponding value is lower for an earlier generation and achieves a smooth increase with the growth of generation, but the growth slope of the matched pixels' curve is more shapely. Contrastively, the value of intensity difference declines gradually if the number of iterations becomes more and more.

### 3.6. Parameter Configuration

Table 1 specifies the parameters used to conduct the experiments for evolving art images. Certainly, it is still an open question how to choose the evolutionary parameters. In the evolutionary art, no human evaluation or interactive evolution is involved.

We list 12 parameters in Table 1, which is divided into four groups, with each group consisting of three parameters. For individual strategy, population size *N*_*p*_, community size *N*_*c*_, and elite ratio *N*_*e*_ are introduced in Section 3.4. For evolution strategy, selection strategy, crossover probability *P*_*c*_, and mutation probability *P*_*m*_ are mentioned in Section 3.5. For update strategy, pixel update method, minimum and maximum circumradius *η*_min_, *η*_max_ are explained in Section 3.3.2. With a large circumradius, the texture is more rough; otherwise, it is fine and smooth. However, too large circumradius should lead to boring feeling, while too small circumradius indicates more white pixels, as shown in Figure 7, and we will further discuss this point. Our evolution process is repeated until a termination condition has been reached. For termination strategy, the terminating conditions include three components. *G*_*m*_ is the maximum generation of the evolution; that is, the evolution is stopped with *t* > *G*_*m*_. *T*_*r*_ is the threshold of pixel ratio, and the system is also terminated when a fixed number of pixels are acceptable. In addition, fitness difference threshold *T*_*f*_ denotes the difference between the highest ranking individual's fitness values of two successive generations, which indicates that the result is reaching or has reached a plateau. In other words, if any one of the above three conditions is satisfied, the proposed algorithm is terminated.

## 4. Experimental Results

### 4.1. Experimental Setup

In this section, several experiments are presented to examine the performance of the proposed technique. We have conducted our experiments on a PC platform with an Intel 2.5 GHz Core i7-6500U CPU and 8 GB RAM, running on a Windows 10 operating system. we describe the experiments in evolving art using various measures and will answer our research questions of this paper by reporting various experimental results with a brief discussion.

### 4.2. Results

#### 4.2.1. Various Strokes

**Figure 6:**
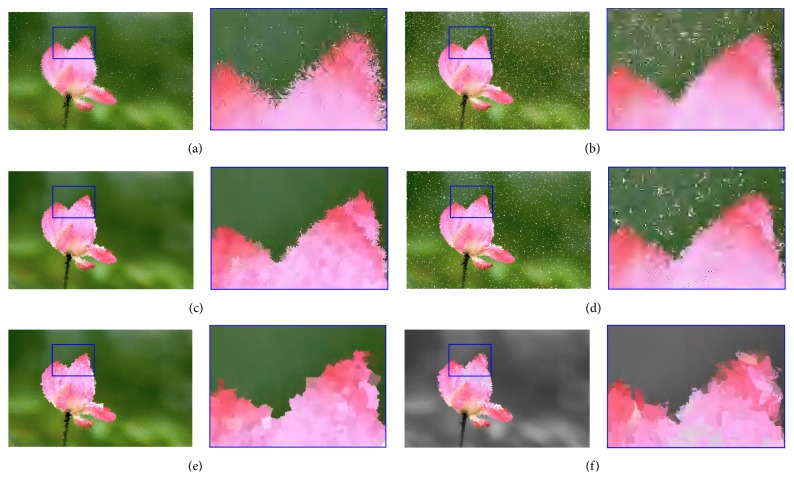
The final results by various strokes. The rendered images for the flower image in Figure 1(a) by (a) the line stroke, (b) the crosshair stroke, (c) the petal-type stroke, (d) the triangle stroke, and (e) the filled square stroke. (f) The rendered image for the flower image in Figure 1(d) by the filled ellipse stroke. Each subfigure is composed of the result and its enlarged portion marked with blue.

The different shapes of strokes can enrich the visual outcome of the proposed system. We investigate six types of strokes according to Algorithm 2 and generate six sample images, including the line, crosshair, petal-type, triangle, square, and ellipse strokes. To make a deep observation, the enlarged details are also attached in Figure 6. Clearly, various strokes would show various styles of result images, and different types of strokes affect the aesthetic qualities of the outputs, which can be potentially applied to various fields. From the perspective of the visual results, the image by the filled square stroke is the most pleasing, since there is no break and no space in the corresponding output. For the reference, six video results (MP4 format) are attached in the Supplementary Materials available online (available here).

#### 4.2.2. Filled Stroke and Empty Stroke

**Figure 7:**
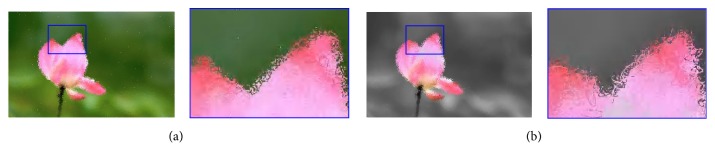
The final results by empty strokes. (a) The rendered image for the flower image in Figure 1(a) by the empty square stroke, (b) the rendered image for the flower image in Figure 1(d) by the empty ellipse stroke. Each subfigure is composed of the result and its enlarged portion marked with blue.

In this subsection, we compare both filled and empty strokes with the shape of square and ellipse to find which one generates more aesthetic pleasing images. Figure 7 shows a comparison of unfilled square and ellipse strokes for the same target image, compared with those by the filled ones in Figure 6. As shown in Figure 7(a), there exist some white pixels or pieces on the canvas, which have not been hit by the empty strokes during the whole evolution, while all of the result pixels have been covered by the filled strokes. At least, one cannot see white pixels in Figure 6 with the naked eye. From a visual perspective, we clearly find the filled strokes achieve better performance and generate more aesthetic images than empty ones. For the reference, two video results (MP4 format) are attached in the Supplementary Materials available online (available here). With the same number of strokes, the filled one obviously covers more pixels; thus white pixels rarely happen. However, this does not mean that the empty stroke will generate bad results. We believe that more individuals can solve this problem by increasing the population size or the community size. However, this should indicate more time costs and more memory spaces, so filled stroke is still recommended considering the performance.

**Figure 8:**
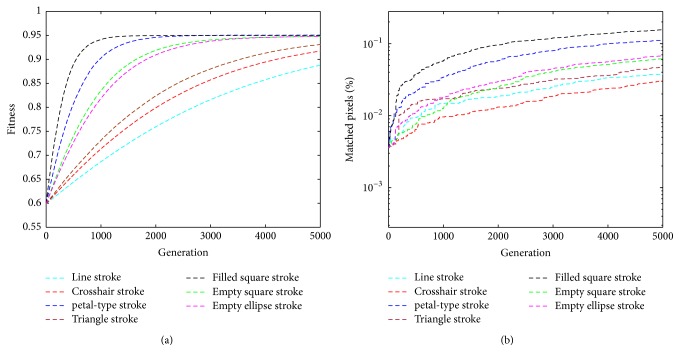
The quantitative results by various strokes in the evolution: (a) the fitness values, and (b) the matched image pixels.

Furthermore, we record the fitness values and the matched image pixels by various strokes within the evolution. For the randomness of the proposed method, we run 50 times for each image and average the results. As shown in Figure 8, we show the progression of fitness values and the matched image pixels during the course of the evaluations. All of the strokes show similar results. For each stroke, both of the curves of fitness values and matched pixels also produce similar tendency with the various generations. With an earlier generation, the fitness values and the matched pixels are very smaller and then increase with the growth of generation. More specifically, the change of the fitness values is more obvious. Intuitively, high fitness values indicate closer resemblance to the target and more matched pixels. In other words, with the growth of generation, the result images gradually reveal the target. Correspondingly, the number of the matched pixels becomes greater and greater. From another perspective, we compare the results by various strokes. Unsurprisingly, although it is subjective to say it, the result images by filled square strokes, at least in our eyes, have better fitness and converge towards a solution faster than the others in the course of iterations. This is because that drawing more pixels in a filled stroke gives more opportunity to be closer to the target. In fact, the covered area by the filled square stroke is the biggest with the same length *R* in Section 3.3.1, followed by that of the petal-type stroke.

#### 4.2.3. Visual Comparison

**Figure 9:**
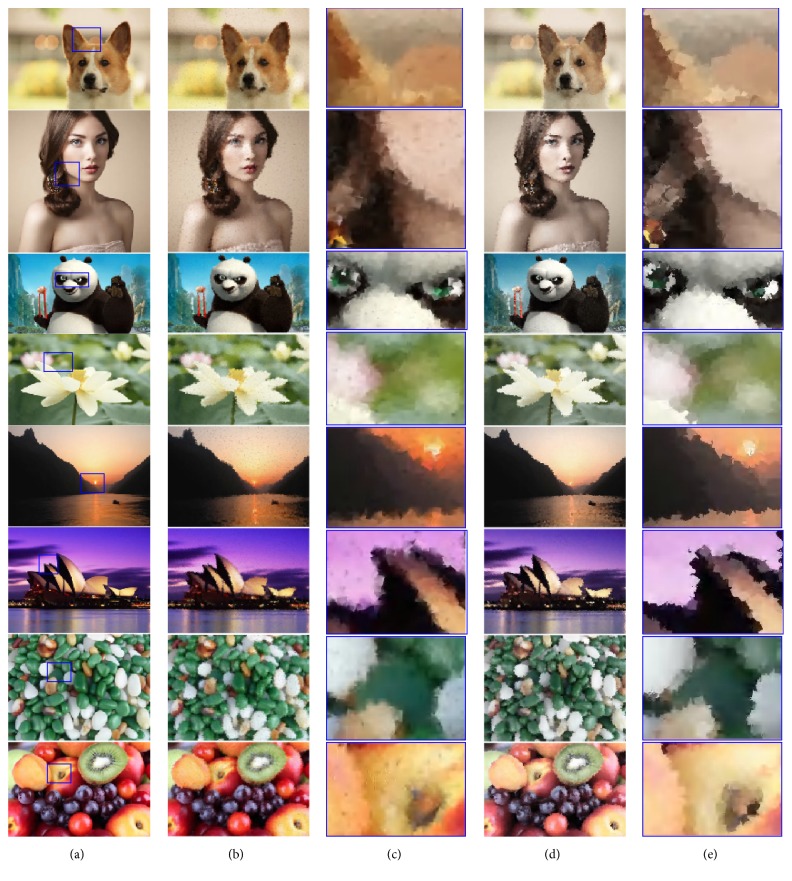
The visual results by various methods: (a) the original image and a subregion marked with blue, the result images by (b) the GIMP method, and (d) our method. (c) An enlarged portion of Figure 9(b) corresponding to the subregion marked with blue in Figure 9(a), (e) an enlarged portion of Figure 9(d) corresponding to the subregion marked with blue in Figure 9(a).

We first examine how the proposed processing technique can benefit the NPR system. Considering that the filled square stroke seems beneficial to fitness scores and the convergence of the evolution, we have experimented with different images using filled square stroke and conducted several runs for each image. As shown in Figure 9, another eight input images of dramatically different scenes (portrait, landscape, seascape, and river land) are chosen for the demonstration out of several runs with different images. Note that to make the paper more compact, we have chosen to shrink all resulting images. However, this should not damage the image quality as each image is associated with a high resolution. Therefore any reader who is interested in seeing more details from an image should always enlarge the corresponding portion.

To show that our rendering technique has indeed good performance, we compare our results with those generated by GNU Image Manipulation Program (GIMP for short) (https://www.gimp.org/), which is a popular, free, and open-source raster graphics editor used for image retouching and editing and more specialized tasks.

The visual comparison results are shown in Figure 9. To make a deep observation, the enlarged details are also listed in Figure 9. For the reference, eight video results (MP4 format) are attached in the Supplementary Materials available online (available here). For these eight images, the rendering results by GIMP method and ours are both visually acceptable. Comparing each result image with the corresponding target, there is the very close visual effect between them, especially when one looks at them from a distance. However, in detail, the result images are covered by one rectangle stroke after another, and these rectangles are located on the canvas with a certain angle, which seem desultory but actually methodical. In other words, a cubism-like style is achieved by the proposed method. From the perspective of visual details, the results by GIMP method show some holes, while our results do not show this flaw, even though we observe them with an enlarge view. The results indicate that our method can be considered as an alternative to the GIMP method.

#### 4.2.4. Quantitative Comparison

**Figure 10:**
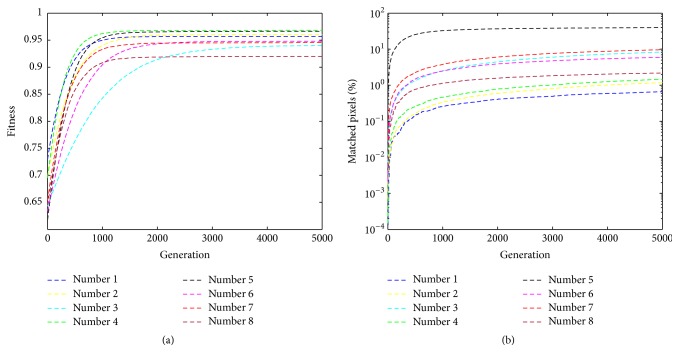
The quantitative results by various images in the evolution: (a) the fitness values, and (b) the matched image pixels.

Furthermore, for these eight images, we record the fitness values and the matched image pixels within the evolution. For the randomness of the proposed method, we also run 50 times for each image and average the results. As shown in Figure 10, we show the progression of fitness values and the matched image pixels during the course of the evaluations. All of these images show similar tendency with the growth of generation. For each image, both of the curves of fitness values and matched pixels grow incrementally as the generation increased one by one, and the increasing degree of the earlier generations is higher than that of the latter. In other words, the cloud model-based evolutionary algorithm is able to find, in all runs and for all images, individuals with high fitness in relatively few generations. In the fitness values, image 4 comes first, and image 3 runs with the slowest convergence. That is to say, the result of Figure 10(a) indicates that the result image 4 is the closest resemblance to the original target and that 3 is the farthest. For the matched pixels, image 5 predominantly achieves the best result, while image 1 always ranks the last. Surely, the image size and the predefined parameters also have an effect on the scores of the matched pixels.

To measure the robustness of the considered methods, we study quantitative scores of the rendering outputs with various images.  Table 2 shows the average response with respect to eight images by using four measures on feature similarity, including the standard pixel-wise peak signal-to-noise ratio (PSNR), mean structural similarity measure (MSSIM) [28], feature similarity (FSIM) [29], and gradient similarity based metric (GSM) [30]. These metrics aim to use computational models to measure the image quality consistently with subjective evaluations, with a full reference image, which is the original input image in this paper, and the distorted image refers to the stylized result.

PSNR index is a traditional image quality assessment metric, and we use it to calculate the peak signal-to-noise ratio for the stylized image with the original image. For PSNR measure, both the GIMP method and ours obtain the highest scores for image 1 as listed in Table 2, but it is noted that image 1 gains the least matched pixels in Figure 10(b). MSSIM index is a dominant method of measuring image quality from perceived change in structural information. For MSSIM measure, the GIMP method receives the highest scores for image 6, but ours for 5, which also ranks the first from the perspective of matched pixels, as shown in Figure 10(b). FSIM index is based on the fact that human visual system understands an image mainly according to its low-level features. For FSIM measure, the best results are still generated by images 6 and 5 using the GIMP method and our method, respectively. GSM index calculates luminance and contrast-structural changes with emphasis on gradient similarity. For GSM measure, most of images produce the higher scores. It means that the two methods well consider the gradients of original images, which convey very important visual information.

On the other hand, the aesthetic performance is a key problem to practical engineering applications of evolutionary art, and it is desired that the evolved images can be generated with a good visual effect. Although the aesthetics problem has a strong subjectivity and the computational aesthetics is one of the most challenging in image processing and computer graphics, we still introduce four common measures as a quantitative evaluation. In this subsection, the aesthetic measures used in our experiments include Benford's law (BL) [31], fractal dimension (FD) [32], global contrast factor (GCF) [33], and Shannon's Entropy (SE) [34]. For full details on these measures, readers are referred to the original papers [31–34]. The results using these four aesthetic measures are also listed in Table 2.

We use Benford's law to measure the distribution of intensity of pixels in result images. BL index calculates the difference between the actual histogram and the Benford histogram. For BL measure, the GIMP method and our method produce similar scores, and both receive a higher value for image 2. FD index reflects the aesthetic preference of people for natural, artificial, and man-made fractals. Images with a higher fractal dimension are considered complex, and those with a lower dimension should be uninteresting. For FD measure, image 1 achieves the highest score using both the GIMP method and ours. GCF index calculates the contrast on various resolutions of an image, and a lower value reflects a lower aesthetic effect. For GCF measure, the two methods gain a higher score for image 7. SE index is also a measure on the intensity of the pixels, and an image will score high on SE if its intensity values are distributed in a uniform way. For SE measure, image 8 always keeps the first rank using the related two methods.

For comparison purposes, each column of the score of each measure is normalized, and then we average the results to provide an overall evaluation. On the whole, our method generates the lower score of the average PSNR. However, it is well known that PSNR does not always agree with the subjective viewing results, particularly for the evaluation of NPR [35]. In addition, our method obtains the smaller average SE value. In other words, our method generates the result images with the intensity of a nun-uniform distribution. For the other six measures, our method is always superior to the GIMP method. To some extent, it is indicated that our method generates better results than the GIMP method, in a couple of respects, that is, image feature similarity and aesthetic performance.

### 4.3. User Evaluation

Unlike photorealistic rendering that can be easy measured by the close degree between the obtained image and the real photo, nonphotorealistic rendering technique is inspired by artistic styles and pays more attention to subjective feelings. For result artworks, different people may have different opinions depending on their artistic quality. So far, each objective index in Table 2 is only a possible reference for measuring the rendering effect of the NPR technique. Therefore, subjective evaluation is a more important complement to assess the nonphotorealistic rendering effect, as well as the assessment of the proposed method.

The objective of the user study is to see if there is a good performance of the proposed method. In the first study, nine testing images are mentioned above and their corresponding rendered results by GIMP and the proposed method are used to perform subjective evaluation. We asked 53 subjects to score each image on the basis of three criteria (image naturalness, image colorfulness, and visual comfort [36, 37]), and these subjects include 48 voluntary undergraduate students with some art knowledge and 5 professional artists. Each score is used a Likert scale in the range [1 10], and 10 means a perfect result.

The result animations by the proposed method are involved into the second study, whose objective is to investigate the performance of the generation process. The second study asked the subjects to score each animation, using a formal protocol designed to answer the following questions: (1) Fidelity. How do you evaluate the fidelity and intuitiveness of these animations? (2) Novelty. Do you find the novel features of them? (3) Interest. Can you gain something interesting from them? (4) Usability. Will you apply them effectively to your future needs? (5) Stability. Is there evidence that they show a uniform style? Each score is also in the range [1 10], and 10 means a perfect result.

**Figure 11:**
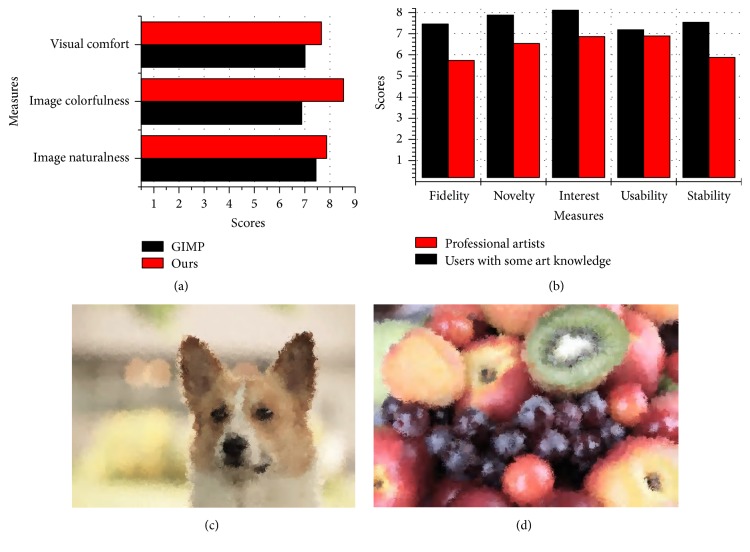
The user study results: (a) on the final image; (b) on the generation process and the corresponding movie; (c), (d) two sample results by the mixed strokes.

Figure 11 shows a summary of the survey results. It can be seen from Figure 11(a) that users generally agree that the proposed method does improve considerably the performance compared with the GIMP method and produces better visual effects. Visual comfort refers to the subjective sensation of comfort [37]. Users rated the visual comfort of the proposed method as 7.6 on a scale of 1 (not comfort) to 10 (very comfort). Image colorfulness presents the degree of color vividness [36]. A rendered image of good quality should have vivid image color. Most of users felt that the proposed method closely mimics the feeling of real artists, while the GIMP method is characterized by artificial footprints. Image naturalness discovers the correspondence degree between human perception and real world [36]. A rendered image of good quality should have natural image color. Our results also obtained better scores. This was due to the fact that the proposed method is based on the reference of the target image.

It can be seen from Figure 11(b) that most of users confirm the performance of the proposed animations. Users commented that they liked the novel look and feel of result animations and would introduce the style into their needs, because of average usability rating 7.2 and 6.9, respectively. In particular, one user commented that the ability to slowly emerge with strokes is the most interesting part. However, as shown in Figure 11(b), the professional artists are generally more critical for the rendering results, giving somewhat lower scores, especially for the fidelity and the stability. In addition, as one user reported, “it has yet to achieve the effect of real painting,” and we believe that this is because the current implementation of the proposed method does not model the type of strokes in evolution population. In fact, an artist never uses only one stroke to paint. Thus, we modify step (9) of Algorithm 1 and randomly select one type of strokes defined in Section 3.3.1, totally 8 strokes, including line stroke, crosshair stroke, petal-type stroke, triangle stroke, and ellipse and rectangle stroke, both empty and filled. The implementation is easy, and only a switch parameter is used to control this function. Then we generate two sample result images as shown in Figures 11(c) and 11(d). One can observe that some new visual effects can be achieved. Compared with the pictures in Figure 9, the texture is more abundant and more complex since we used the mixed strokes to model the style of a real artist. Anyway, another possible fun can be provided by the proposed method. According to the user study, it is also noted that the proposed method is more suitable as a technique to serve the general public, rather than a professional art tool. In general, we believe that the approach can be a good alternative to GIMP for novice users, to convert their photos into NPR style, though it is not very powerful in its current state.

### 4.4. Discussions

In this subsection, we provide a brief discussion on the proposal. Relating to research question (1) in Section 1, we propose a framework for the image-guided rendering using a cloud model-based evolutionary algorithm, which can be divided into four parts, including image feature extraction (i Phase), strokes-based genetic representation and rendering (R Phase), cloud model-based evolutionary knowledge (C Phase), and cloud model-based evolutionary engine (E Phase). For the reference of the proposed framework, a recommended parameter configuration is also proposed. Our method involves a novel algorithm using cloud model-based evolutionary. In addition, the proposed fitness reproduction is an uncertain measure using the forward and backward normal cloud generator, and the overall procedure is an automatic evaluation, but not directly by user interaction. Relating to research question (2), we provide six different types of strokes in Section 3.3.1 and investigate the aesthetic qualities of the rendered result images. The experimental results show that the different shapes of strokes can enrich the visual outcome of the proposed system, and the filled square strokes achieve better performance and generate more aesthetic images from the perspective of the visual results. Furthermore, the temporary image for each generation produces a dynamic result by iterations, which is a much better fit to the user's needs. In addition, the proposed method has an immersion effect because it can provide the users with the process of generating images, that is the animations.

## 5. Summary and Conclusion

In the paper, we propose an algorithm of evolving art using cloud model. Furthermore, we introduce six types of strokes into the proposed framework of evolutionary art, both filled and empty. The proposed framework can generate interesting images and animations with the NPR style based on a given input image. The resulting animations have an interesting characteristic in which the target slowly emerges from a set of strokes. The experiment results, including visual comparisons, quantitative comparisons, and user studies, verify the efficiency and feasibility of the proposal, which can be considered as an alternative for novice users to the existing methods, to convert their photos into NPR style, for example, the GIMP method.

There are a couple of issues that will be considered in the future research: (1) As mentioned by [4], introducing other fitness functions to explore the new visual effect of evolved images, especially for more pleasuring texture, is currently under investigation and will be reported later; (2) More alternative evolutionary algorithms based on cloud model may be provided just as or more interesting than result images; thus the extension of the proposed method to improve the performance is well worth further studying on, for example, introducing computational ecosystems [38].

## Figures and Tables

**Algorithm 1:**
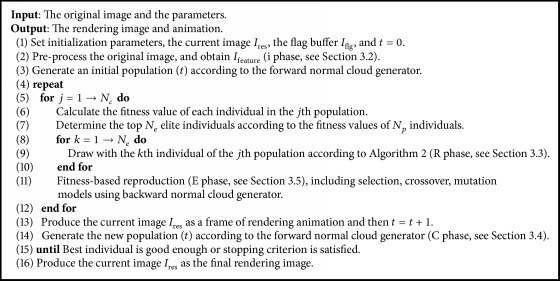
The iREC algorithm.

**Algorithm 2:**
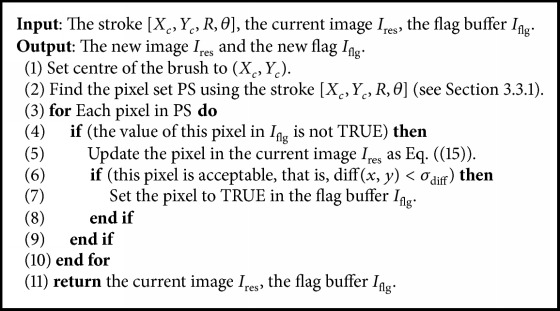
The rendering algorithm.

**Table 1:** Parameter configuration.

Type	Name	Parameter	Value	Default
Individual strategy	Population size	*N*_*p*_	10–100	40
	Community size	*N*_*c*_	3–10	5
	Elite ratio	*N*_*e*_	3–100	10

Evolution strategy	Selection	/	Roulette wheel	
	Crossover probability	*P*_*c*_	0.5–0.95	0.6
	Mutation probability	*P*_*m*_	0.001–0.15	0.1

Termination strategy	Fitness difference threshold	*T*_*f*_	0.0001–0.01	0.0005
	Maximum generation	*G*_*m*_	3000–10000	5000
	Pixel ratio threshold	*T*_*r*_	0.2–0.6	0.5

Update strategy	Maximum circumradius	*η*_max_	2–100	10
	Minimum circumradius	*η*_min_	1–20	3
	Pixel update method	/	Replace, blend, or hold	Blend *ω* = 0.3

**Table 2:** Quantitative evaluation results.

Images	Methods	PSNR	MSSIM	FSIM	GSM	BL	FD	GCF	SE
Number 1	GIMP	37.515	0.815	0.876	0.988	0.958	0.767	3.155	7.689
	Ours	35.847	0.878	0.909	0.992	0.965	0.789	3.049	7.518
Number 2	GIMP	36.723	0.820	0.887	0.987	0.988	0.613	4.036	7.693
	Ours	35.504	0.876	0.913	0.990	0.984	0.638	4.032	7.599
Number 3	GIMP	37.027	0.773	0.870	0.986	0.985	0.531	5.209	7.893
	Ours	33.924	0.772	0.863	0.986	0.984	0.503	5.444	7.859
Number 4	GIMP	33.723	0.825	0.878	0.986	0.954	0.613	4.654	7.777
	Ours	33.040	0.871	0.887	0.988	0.957	0.627	4.613	7.681
Number 5	GIMP	36.354	0.810	0.863	0.988	0.976	0.462	4.817	7.206
	Ours	34.337	0.886	0.923	0.992	0.982	0.488	4.722	7.066
Number 6	GIMP	35.471	0.831	0.892	0.987	0.980	0.419	5.734	7.817
	Ours	33.540	0.847	0.893	0.987	0.984	0.449	5.738	7.837
Number 7	GIMP	31.788	0.738	0.826	0.976	0.977	0.443	8.119	7.849
	Ours	30.223	0.751	0.818	0.975	0.977	0.444	8.407	7.848
Number 8	GIMP	33.396	0.742	0.856	0.982	0.946	0.534	6.247	7.905
	Ours	29.099	0.724	0.824	0.979	0.947	0.588	6.314	7.875

Average	GIMP	**0.731**	0.434	0.483	0.569	0.585	0.348	0.410	**0.790**
	Ours	0.486	**0.628**	**0.579**	**0.640**	**0.630**	**0.397**	**0.418**	0.708

## Supplementary Materials

Supplementary Materials Supplementary Data on Video Results: supplementary data associated with this article can be found online at the following site: https://1drv.ms/f/s!AjY7hx6hoejqcqrb5MogLAtVUUA. In the Supplementary Materials, we provide four “7-zip” archiver files, which include various video results with MP4 format, mentioned in Figures 5, 6, 7, and 9, respectively.
